# Functionalized, Vertically Super-Aligned Multiwalled Carbon Nanotubes for Potential Biomedical Applications

**DOI:** 10.3390/ijms21072276

**Published:** 2020-03-25

**Authors:** Patrick P. Komane, Pradeep Kumar, Yahya E. Choonara, Viness Pillay

**Affiliations:** 1 Wits Advanced Drug Delivery Platform Research Unit, Department of Pharmacy and Pharmacology, School of Therapeutic Sciences, Faculty of Health Sciences, University of the Witwatersrand, Johannesburg, 7 York Road, Parktown 2193, South Africa; patrickk@uj.ac.za (P.P.K.); pradeep.kumar@wits.ac.za (P.K.); yahya.choonara@wits.ac.za (Y.E.C.); 2 Department of Chemical Sciences, University of Johannesburg, 27 Nind Street, Doornfontein, Johannesburg 2028, South Africa

**Keywords:** chemical vapor deposition, multiwalled carbon nanotubes, silicon wafer, contact angle, PEGylation, nanoelectrode

## Abstract

Currently, there is a lack of ultrasensitive diagnostic tool to detect some diseases such as ischemic stroke, thereby impacting effective and efficient intervention for such diseases at an embryonic stage. In addition to the lack of proper detection of the neurological diseases, there is also a challenge in the treatment of these diseases. Carbon nanotubes have a potential to be employed in solving the theragnostic challenges in those diseases. In this study, carbon nanotubes were successfully synthesized for potential application in the detection and treatment of the neurological diseases such as ischemic stroke. Vertically aligned multiwalled carbon nanotubes (VA-MWCNTs) were purified with HCl, carboxylated with H_2_SO_4_:HNO_3_ (3:1) and acylated with SOCl_2_ for use in potential targeting studies and for the design of a carbon-based electrode for possible application in the diagnosis of neurological diseases, including ischemic stroke. MWCNTs were washed, extracted from the filter membranes and dried in a vacuum oven at 60 °C for 24 h prior to functionalization and PEGylation. CNTs were characterized by SEM, TEM, OCA, DLS, CV and EIS. The HCl-treated CNT obtained showed an internal diameter, outer diameter and thickness of 8 nm, 34 nm and 75 µm, while these parameters for the H_2_SO_4_-HNO_3_-treated CNT were 8 nm, 23 nm and 41µm, respectively. PEGylated CNT demonstrated zeta potential, polydispersive index and particle size distribution of 6 mV, 0.41 and 98 nm, respectively. VA-MWCNTs from quartz tube were successfully purified, carboxylated, acylated and PEGylated for potential functionalization for use in targeting studies. For designing the carbon-based electrode, VA-MWCNTs on silicon wafer were successfully incorporated into epoxy resin for diagnostic applications. Functionalized MWCNTs were nontoxic towards PC-12 neuronal cells. In conclusion, vertically super-aligned MWCNTs have been successfully synthesized and functionalized for possible theragnostic biomedical applications in neurological disorders such as ischemic stroke.

## 1. Introduction

Neurological disorders pose a huge burden on global health. Brain is the most vital organ of human beings that is protected by the blood-brain barrier (BBB) to restrict the access of harmful substances from the peripheral blood circulation. However, the BBB also restricts delivery of therapeutics into the diseased site of the brain, making treatment of central nervous system (CNS) disorders a most difficult task. Stroke is one of the neurological diseases and is regarded as the fifth root cause of death and the primary cause of permanent disability globally. Ischemic stroke contributes 80%, and haemorrhagic stroke makes up the remaining 20% [1]. There is a dire need for effective diagnosis and treatment of stroke at the present moment. Nanotechnology in medicine is an emerging field with a possible application of the engineered nanomaterials that could be implemented in the treatment of diseases, including neurological disorders. 

Carbon nanotubes (CNTs) are promising smart nanomaterials for the advancement in technology based on their unique architecture and outstanding physico-chemical properties; in particular, high surface area, small size, nonimmunogenicity, cell membrane penetrability, size alterability, nontoxicity, photostability, ultralight weight and biocompatibility. CNTs have a plethora of potential applications in different fields of nanomedicine [2]. Currently, more attention has been directed to carbon nanotubes as a result of their nanosize, electronic, optical, thermal, electrical, mechanical and chemical characteristics [3]. These unique properties of CNTs make them desirable as a material for use in biomedical applications such as detection and treatment of neurological diseases, including ischemic stroke. Diagnosis and treatment of neurological diseases such as ischemic stroke are extremely challenging because of the blood–brain barrier (BBB). This barrier obstructs the access to the brain for diagnosis and delivery of the drugs to the diseased site. CNTs have been demonstrated to have a potential to overcome these challenges. Kafa and co-workers, 2015, successfully delivered multiwalled carbon nanotubes (MWCNTs) to the mouse brain (animal model of Alzheimer’s disease) following functionalization and conjugation to the Aβ binding molecule [4]. 

Carbon nanotubes have been used in several biomedical and biosensing applications involving (a) an immunosensor for detection of T-2 mycotoxins in feed and pork [5], (b) a biosensor for detection of 17β-estradiol in humans [6], and (c) a electrochemical sensor for detection of uric acid in blood and urine [7]. In addition, MWCNTs when stereotactically delivered to the mouse brain were found to reduce blood pressure drastically in the treated mouse [8]. Furthermore, carbon nanotube templates were used to effectively guide human neurite stem cell growth, and this mechanism may lead to improved therapeutic effects in the injured spinal cord [9]. 

Pristine carbon nanotubes are hydrophobic in nature and, thus, insoluble in water. They have a tendency to agglomerate due to their chemical inertness and van der Waals forces on their surfaces. This property creates an obstacle in their biomedical applications, and this may lead to toxicity. As a result, a variety of functionalization methods have been developed in order to facilitate dispersion in an aqueous milieu to enable their application in a physiological environment [10]. 

There are two procedures that are commonly employed in functionalization of carbon nanotubes, namely noncovalent and covalent functionalization. Carbon nanotubes are coated with hydrophilic molecules in noncovalent functionalization, whereas the functional groups are attached to the backbone of carbon nanotubes in covalent functionalization. Functionalization of carbon nanotubes has been found to reduce their cytotoxicity. Therefore, carbon nanotubes are considered to be the smart nanomaterials of the 21st century, with a wide spectrum of applications in diverse fields, including biomedical, tissue engineering, biosensor technology and drug delivery [11]. 

In this study, vertically super-aligned MWCNTs were synthesized using chemical vapor deposition (CVD). Vertically aligned CNTs were manufactured for the development of the nanoelectrode for diagnosis and for the development of the nanocarrier to deliver drugs to the diseased site in neurological disorder such as ischemic stroke. CNTs were purified with HCl, carboxylated with H_2_SO_4_:HNO_3_ and acylated with SOCl_2_. Their inertness and hydrophobicity were drastically changed following purification and functionalization. They became highly hydrophilic, electrically conductive, highly stable and reduced in size for use in biomedical application. Vertically aligned (VA)-MWCNTs grown on silicon wafer were incorporated in epoxy resin for the development of a system for application in the diagnosis of neurological disorders such as ischemic stroke. In our study, we have successfully synthesized and functionalized vertically super-aligned multiwalled carbon nanotubes for the development of nanoelectrodes for the diagnosis of diseases and future applications in the treatment of a vartiety of neurological diseases, including ischemic stroke. 

## 2. Results and Discussion

### 2.1. Box-Behnken Design of Experiments (BBDOE)

Box-Behnken design of experiments was performed to synthesise VA-MWCNTs with specific thickness, external diameter and internal diameter in both the quartz tube and on the substrate. This design was employed to obtain the optimal pyrolysis conditions for the successful VA-MWCNT growth with those features of interest, with the main focus on the three variables: namely, synthesis temperature, gas flow rate and synthesis time. 

MWCNTs were produced on wafer with max mass 11.80 mg, max length 270.33 µm, max internal diameter 9.67 nm and max external diameter 39.67 nm, when the following pyrolysis conditions were applied: reaction duration = 45 min, carrier gas flow rate = 400 mL·min^−1^ and reaction temperature = 775 °C (Table S1). MWCNTs were also produced on a quartz tube wall with max mass 1446.95 mg, max length 181.83 µm, max internal diameter 12.13 nm and max external diameter 45.17 nm. The pyrolysis conditions were as follow: reaction duration = 60 min, flow rate of gas = 200 mL·min^−1^ and reaction temperature = 900 °C (Table 2). 

### 2.2. Aligned MWCNTs and Treatment with the Acids

**Figure 1:**
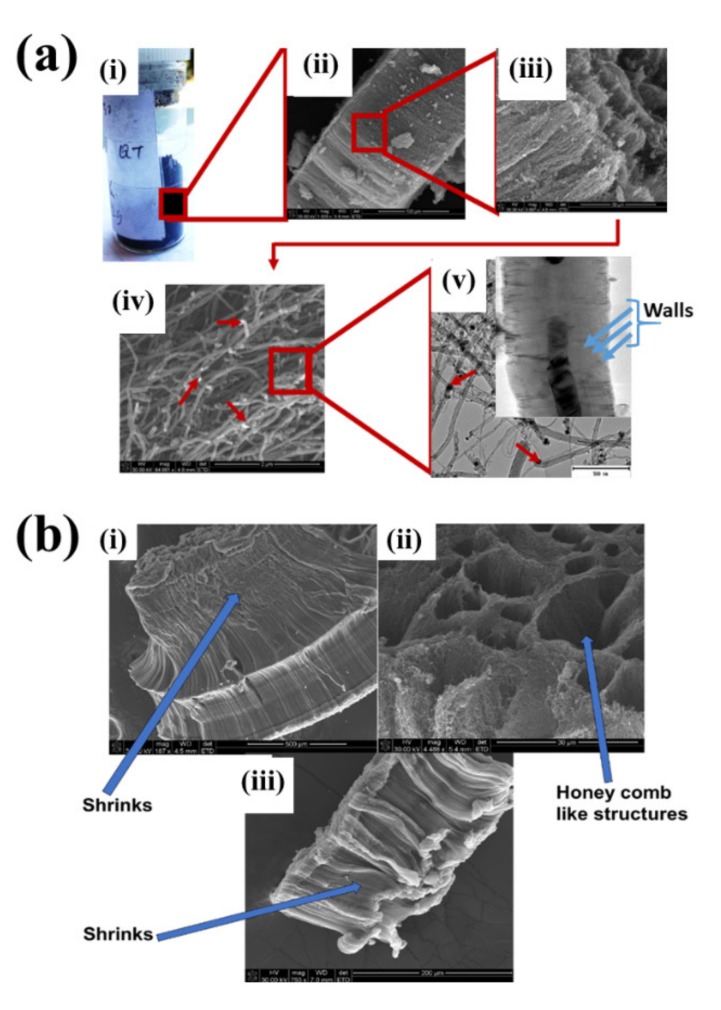
Multiwalled carbon nanotubes (MWCNTs) grown on quartz tube and acid treatment. CNTS collected from quartz tube wall (**a**(i)), SEM micrograph of CNTs at 1005× magnification (**a**(ii)), SEM micrograph of CNTs at 3697× magnification (**a**(iii)), SEM micrograph of CNTs at 64,991× magnification (**a**(iv)) and TEM micrograph of CNTs with an insert at very high magnification with blue arrows indicating the CNT walls (**a**(v)). Conc. H_2_SO_4_:HNO_3_ (3:1) (**b**(i)), 5 M HCl (**b**(ii)) and Conc. HCl (**b**(iii)).

CNTs collected from the quartz tube walls were vertically aligned but not as perfect and clean as the Si wafer ones. The quartz tube wall CNTs were contaminated with amorphous carbon and Fe nanoparticles, as seen in Figure 1a(i,ii). Alignment and contamination were further confirmed when magnification was increased from 1005 to 3697× in Figure 1a(iii). As seen in Figure 1a(iv) (see red arrows), alignment was clear, and most of the tips had Fe nanoparticles as opposed to Si wafer CNTs. TEM micrograph revealed the contamination by amorphous carbon and Fe nanoparticles, as depicted in Figure 1a(v). 

CNTs were also having more than one wall with Fe nanoparticles embedded in the central cavity of the CNT, as demonstrated in Figure 1a(v) (see insert). Contamination from amorphous carbon and Fe nanoparticles was due to the variation of temperature in different zones of the quartz tube. It was also due to the variation of the gas flow rate as the gas travels in different temperature zones within the tube. Since there was a temperature variation within the tube, this could also lead to variation in the evaporation rate of the catalyst. Our data has demonstrated that there are more contaminants in the quartz tube wall grown CNTs compared to Si wafer CNTs. Since two different substrates were used, the difference is expected as there are different degrees of interaction between the CNTs and the substrates. 

An exposure of VA-MWCNTs to conc. HNO_3_:H_2_SO_4_ (1:3) caused a deformation on the CNTs. The CNTs shrank and formed a cone-like structure with two layers, as seen in Figure 1b(i). A mixture of HNO_3_ and H_2_SO_4_ oxidized the iron nanoparticle and amorphous carbon layers. However, when VA-MWCNTs were exposed to 5-M HCl, honeycomb-like structures with mean diameters of 10.5 µm were formed on the surface of the CNTs, as demonstrated in Figure 1b(ii). Concentrated HCl resulted in the shrinkage of the CNTs illustrated in Figure 1b(iii). 

Quyen and colleagues synthesized MWCNTs from LPG on Fe_2_O_3_/Al_2_O_3_ precatalyst via CVD without hydrogen. CNTs with less defective structures were obtained with identical external diameters of 50 nm [12]. In our study, aligned CNTs were produced when ferrocene was used as a catalyst. As-synthesized CNTs were hydrophobic and developed honeycomb-like structures when treated with HCl and a mixture of H_2_SO_4_:HNO_3_ (3:1), which made them hydrophilic. 

### 2.3. Carboxylation, Acylation and Morphological Evaluation of MWCNTs

VA-MWCNTs were carboxylated and acylated to attach functional groups to improve coupling with other molecules of interest. MWCNTs were refluxed with conc. HCl and HNO_3_ to remove carbon and iron prior to further treatment. MWCNTs were refluxed with H_2_SO_4_: (3:1 ratio) to attach COOH to generate MWCNT-COOH for further functionalization. MWCNTs were refluxed with SOCl_2_ in DMF to add acyl group to generate MWCNT-COCl (more reactive) for further functionalization. 

VA-MWCNTs were then coated with Pd/Au prior to scanning with SEM for morphological evaluation and size determination. For TEM scanning, CNTs were dispersed in ethanol for determination of external and internal diameters. It was found that functionalization of the MWCNTs with HCl and H_2_SO_4_:HNO_3_ reduced the thickness or length of the MWCNTs. A greater reduction in the thickness of the CNTs was observed in the H_2_SO_4_:HNO_3_-treated CNTs than in the HCl_-_treated CNTs (Table 1). 

The external diameters of the MWCNTs were also drastically decreased in the H_2_SO_4_:HNO_3_-treated CNTs, as depicted in Table 1. This is indicative that sulphonitric acid has a stronger oxidizing strength than HCl. Taklimi and colleagues observed minor modifications, such as reduction in length, size and shape, when helical MWCNT were exposed to sulphonitric solution H_2_SO_4_:HNO_3_ [13]. On average, the internal diameters remained constant around 10 nm. 

The internal diameter was expected to remain constant, as it is dependent on the size of the Fe nanoparticles (10 nm) used for the formation of nucleation sites in the synthesis of the CNTs [14]. The sulphonitric acid-treated MWCNTs have demonstrated some deformation of the surface and individual CNTs, as depicted in the TEM micrographs in Table 1. The individual CNTs were not morphologically distinguishable, and this made it difficult to identify them with conformity. 

### 2.4. Dispersity, Particle Size Distribution and Zeta Potential of MWCNTs

**Figure 2:**
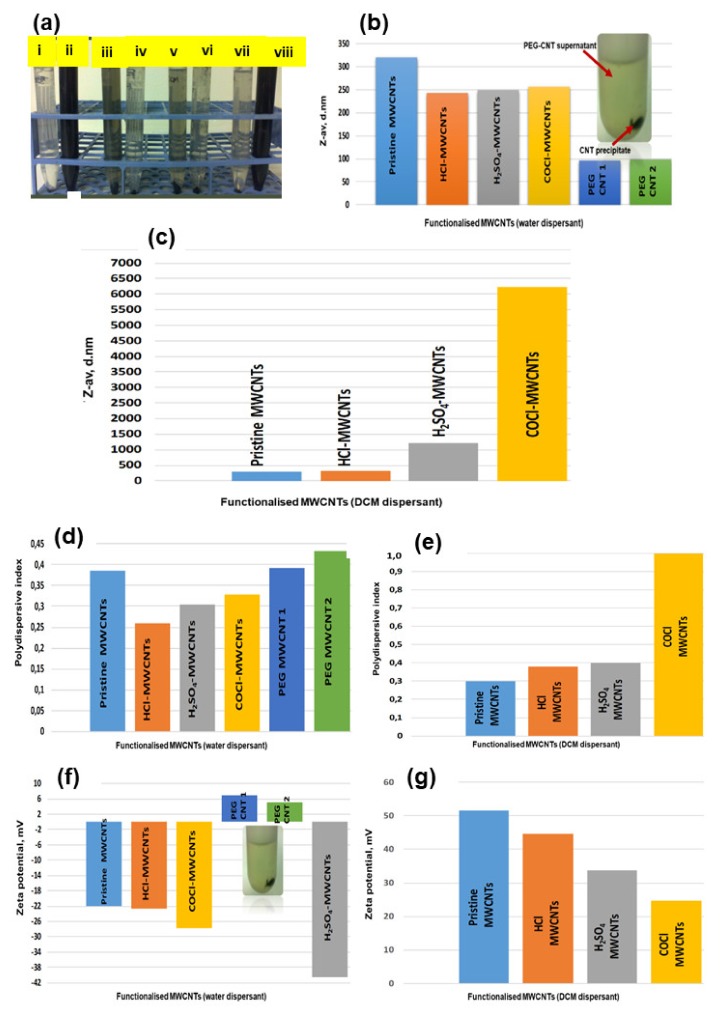
Dispersibility of pristine and functionalized MWCNTs in water and dichloromethane (DCM). COCl-CNTs in DCM (**a**)i, COCl-CNTs in water (**a**)ii, CNTs in DCM (**a**)iii, pristine CNTs in water (**a**)iv, HCl-CNTs in DCM (**a**)v, HCl-CNTs in water (**a**)vi), H_2_SO_4_-CNTs in DCM (**a**)vii and H_2_SO_4_-CNTs in water (**a**)viii. Particle size distribution (PSD) of CNTs dispersed in water (**b**), CNTs dispersed in DCM (**c**), Polydispersive index (PDI) of functionalized CNTs dispersed in water (**d**) and in DCM (**e**). Zeta potential of CNTs dispersed in water (**f**) and in DCM (**g**).

CNT dispersibility differs from dispersant to dispersant as a result of their physico-chemical structures. MWCNTs functionalized with carbonyl chloride were readily dispersed in water than in dichloromethane (DCM), as demonstrated by a dark solution in Figure 2a(ii) and a crystal clear solution in Figure 2a(i). COCl-CNTs in DCM dispersant sedimented at the bottom of the tube, implying that there was no interaction of the CNTs with the DCM, as illustrated in Figure 2a(i). 

DCM is less polar than water, as it has a polar index of 3.1, and water has a polar index of 10. Therefore, water is the most polar solvent compared to other solvents. Pristine MWCNTs were more moderately dispersed in DCM than in water due to their hydrophobic nature. There was a minor dispersion in water, as demonstrated by a moderate change in color from clear to a light dark color in Figure 2a(iii,iv). 

HCl-CNTs were more moderately dispersed in DCM than in water but with a slight increase in intensity in water compared to pristine CNTs in the water above, as demonstrated in Figure 2a(v,vi). In H_2_SO_4_-CNTs in DCM, the solution was crystal clear, which implies that there was no interaction or there was a very slight unnoticeable interaction of the CNTs with DCM, as shown in Figure 2a(vii). H_2_SO_4_-CNTs dispersed readily in water dispersant, as demonstrated in Figure 2a(viii). These findings have demonstrated that MWCNTs functionalized with carbonyl chloride (SOCl_2_) and H_2_SO_4_ are highly dispersible in water and, hence, highly reactive. 

Dynamic light scattering spectroscopy provides information on the size distribution of nanomaterials [15]. This technique is most suitable for the determination of diameter of spherical particles, but it is still applicable for measuring hydrodynamic diameters of nanotubes [16]. Zeta potential is based on electrophoretic mobility for charge determination on the particles. Zeta nanosizer was used to measure both the zeta potential and particle size distribution of the CNTs [17]. 

Pristine MWCNTs dispersed in water had a higher particle size than MWCNTs dispersed in DCM, as shown in Figure 2b,c and Table 2. 

Individual MWCNTs in their native state have a tendency of agglomerating to each other; hence, agglomerates with bigger hydrodynamic diameters are formed. Pristine MWCNTs particles were readily dispersed in DCM than in water due to their hydrophobic nature, as confirmed by their inability to disperse readily in water, as demonstrated in Figure 2a(iii,vi). 

Water is a polar dispersant, whereas DCM is 3x less polar than water. Pristine CNTs are hydrophobic; hence, they disperse readily in DCM, as confirmed by a reduction in particle size illustrated in Figure 2c. 

COCl-MWCNTs are hydrophilic and will not disperse in hydrophobic dispersant, as shown by the increase in particle size due to the agglomeration of the particles (Figure 2c). Functionalization of CNTs with HCl, H_2_SO_4_, COCl and PEG changes the hydrophobic nature of CNTs to a hydrophilic nature, and this improves the dispersibility of the CNTs in water. As the dispersibility is increased, the agglomerates are broken down, and particle size of the CNTs is reduced in water, as shown in Figure 2b,c and Table 2. 

In PEGylated CNTs, the particle size dropped drastically as the PEGylated CNTs were readily dispersed in water. The unreacted and bigger particles of the CNTs were sedimented at the bottom of the tube, and the supernatant rich in PEG-CNTs were used in this dispersibility study, as illustrated in Figure 2b. 

Polydispersive index (PDI) is used to determine the particle distribution type. It is a measure of heterogeneity of sizes of molecules or particles in a mixture. The distribution is uniform if the particles have the same size and shape. The uniform distribution has a PDI = 0.0, narrow distribution PDI = 0.0–0.1, moderate distribution PDI = 0.1–0.4 and broad distribution >0.4. 

In this work, PDI of pristine MWCNTs dispersed in DCM was found to be 0.3 and, in water, was 0.39. According to these findings, pristine MWCNTs distribution type was moderate in both water (PDI < 0.4 > 0.3) and DCM. PDI of pristine MWCNTs was found to be high in water than in DCM (Figure 2d,e and Table 2). The HCl-MWCNTs had PDIs less than 0.4 in both the water and DCM, which implied that the distribution was moderate. The PDI of HCl-treated MWCNTs was 0.26 and 0.38 in both water and DCM, respectively, as depicted in Figure 2d,e and Table 2. 

In H_2_SO_4_, functionalized MWCNTs dispersed in water; the particle size was very small around 253 nm, as opposed to DCM, which was around 1223 nm, as demonstrated in Figure 2b,c. MWCNTs in DCM agglomerated, hence, a bigger diameter and readily dispersed in water, as illustrated in Figure 2b,c. The PDI of H_2_SO_4_ functionalized MWCNTs in water and DCM were 0.344 and 0.400, respectively, hence, a moderate particle distribution, as shown in Figure 2b,c. 

MWCNTs functionalized with SOCl_2_ dispersed in water had a smaller size, as opposed to the MWCNTs dispersed in DCM with diameters of 256 nm and 6220 nm, respectively, as illustrated in Figure 2a(ii,iii). These CNTs dispersed readily in water compared to DCM, as shown in Figure 2a(i,ii). PDI for COCl-CNTs dispersed in water was 0.330 and, in DCM, was 1.00, respectively (Figure 2d,e). CNTs distribution type was moderate for water-dispersed and very broad for DCM-dispersed based on the results obtained. Particle sizes in the PEG-MWCNTs in water were very small (97 nm–99 nm) compared to the pristine, carboxylated and acylated MWCNTs, as demonstrated in Figure 2b. The PEGylated CNT with smaller sizes were dispersed in the solution, and those with bigger sizes were unreacted with PEG sedimented at the bottom of the centrifuge tube, as observed in Figure 2b (see insert). The PDI was around 0.4, as observed in Figure 2d, which implied that the PEGylated CNTs had a moderate distribution type. It can therefore be concluded from our findings that acylation increases PDI of MWCNTs in DCM. 

Zeta potential (ZP) gives the magnitude of the charge repulsion/attraction between the particles. It is a physical property exhibited by any particle in dispersion. The ZP magnitude can be used in the dispersion system as an indication of the aqueous stability [18]. If all particles in dispersion possess a large negative or positive ZP, then they tend to repel each other. There will be no tendency for the particles to agglomerate. If particles have low ZP, then there will be no force to hinder the particles to coagulate. Zeta potential distribution types are as follows: rapid coagulation ZP = 0–10 mV, incipient instability ZP = 10–30 mV, moderate stability ZP = 30–40 mV, good stability ZP = 40–60 mV and excellent stability. 

Pristine, carboxylated and acylated MWCNTs dispersed in water were found to have a negative ZP and those dispersed in DCM, positive ZP (Figure 2f,g). PEGylated MWCNTs dispersed in water were positively charged with small ZP (+5 mV to +7 mV). ZP of pristine MWCNTs in water and DCM were found to be −22 mV and +52 mV, respectively (Figure 2f,g). Zeta potential in the carboxylated and acylated MWCNTs dispersed in DCM were higher than in water, which implies that they are more stable in DCM compared to water. 

These findings have demonstrated that the carboxylation, acylation and PEGylation of the CNTs dispersed in water reduced the particle size, and the particle distribution was found to be moderate. ZP was negative in CNTs dispersed in water and positive in CNTs dispersed in DCM. Functionalization improved the stability of the CNTs, with a higher impact in CNTs dispersed in DCM. 

### 2.5. Elemental Composition and Effect of Gas Flow Rate on the Yield of VA-MWCNTs

**Figure 3:**
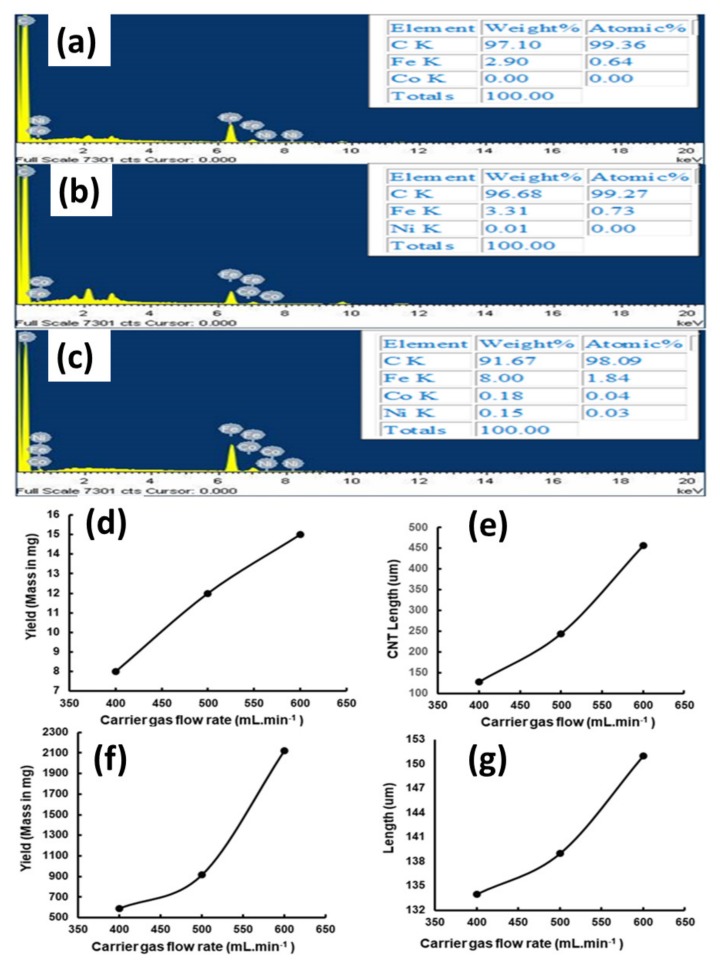
Elemental composition and yield of MWCNTs. Fe-Co (**a**), Fe-Ni (**b**) and Fe-Co-Ni (**c**). CNT yield on silicon wafer (**d**), CNT length on silicon wafer (**e**), CNT yield from quartz tube walls (**f**) and CNT length from quartz tube walls (**g**).

SEM-EDS was used to determine the elemental composition of MWCNTs in this study. The results show the presence of carbon and iron, since MWCNTs contain carbon from toluene and iron from ferrocene in the catalyst. Co has been detected but at a very low level, which could not be quantified (Figure 3a). The concentration of Co in the catalyst solution containing Co and ferrocene in toluene was far much lower than the limit of detection (LOD) of the FEI Nova NanoLab 600 FEG-SEM/FIB. Ni was not detected in these CNTs, as it was not included in the catalyst solution. 

Nickel (Ni) was detected but at a very low level when the Fe-Ni catalyst (bimetallic catalyst) was used for CNT synthesis. Co was not detected in the CNTs, as it was not included in the catalyst. Fe and carbon were detected, as their sources were used to prepare the catalyst (Figure 3b). Fe, Ni, Co and carbon were detected in the MWCNTs, synthesized using a ferrocene-nickelocene-cobaltocene catalyst (trimetallic catalyst). Ni and Co were detected in less amounts as a result of overcoating by iron (Figure 3c). These results are indicative that the vertically aligned MWCNTs were successfully synthesized using bimetallic and trimetallic catalysts. 

A change in the flow rate of the carrier gas has an impact on the structure and morphology of carbon nanotubes. When a mixture of gases contacts the catalyst surface, an equilibrium is achieved between the molecules in the gaseous phase and the adsorbed species on the catalyst. 

There will not be any synthesis of CNTs when there is no flow of carrier gas to deliver the catalyst into the reactor. CNT synthesis is initiated when there is a flow of carrier gas and the yield and length increase until the gaseous molecules and adsorbed species bound to the catalytic surface reach an equilibrium. The yield is negatively affected, as the flow rate is too high; hence, the formation of amorphous carbon is observed. 

In our study, various argon flow rates from 400 to 600 mL·min^−1^ were used, and a direct linear relationship was observed between the flow rate of the carrier gas and thickness and yield of carbon nanotubes. The yield and thickness of CNTs increased with a rise in the flow rate of carrier gas, as depicted in Figure 3d–g. The flow rate of the carrier gas had an impact on the deposition of the carbonaceous products on the reactor walls and silicon wafer. It can be concluded that an increase in the flow rate of the carrier gas led to an increase in the CNT yield and length. 

### 2.6. Crystalinity of VA-MWCNTs

**Figure 4:**
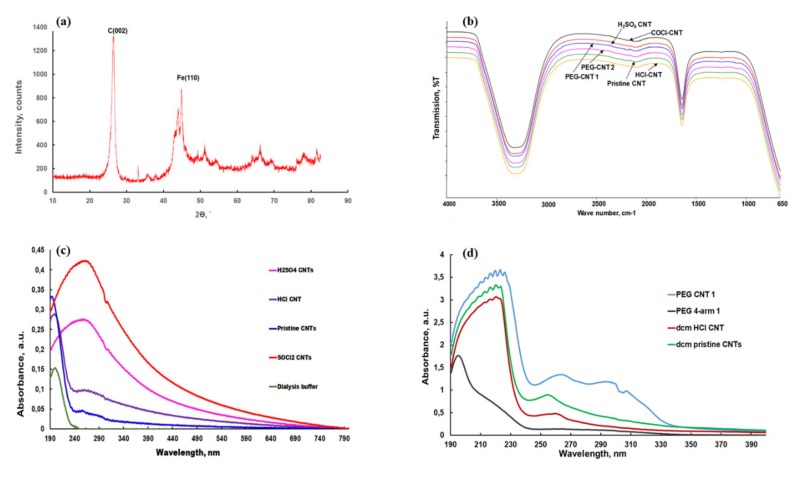
Si wafer XRD and IR spectra of VA-MWCNTs. XRD spectrum of Si wafer (**a**), universal attenuated total reflectance (UATR)-IR spectra of CNTs in water (**b**), UV-Vis spectra of carboxylated MWCNTs (**c**) and UV-Vis spectra of the PEGylated MWCNTs (**d**).

The XRD was performed on CNTs grown on Si wafer prior to nanoelectrode development. Two characteristic peaks were observed at the angle (2θ) of 25° and 45°, as demonstrated in Figure 4a. The peaks for the as-synthesized MWCNTs arrays on Si wafer indexed to (002) and (110) at 2θ of 25° and 45° are indicative of the presence of hexagonal graphite (carbon) and catalytic impurities (iron) respectively. 

The Fe peak has less intensity as compared to C peak, and this observation could be due to the fact that the CNTs were purified and oxidized, leading to a reduction in Fe content in the as-synthesized CNTs. 

### 2.7. IR and UV-Vis Spectra of MWCNTs

FTIR was performed to assess the functional groups coupled to MWCNTs following functionalization with acids and carbonyl chloride. Pristine MWCNTs showed important absorption bands at 3444.6 cm^−1^ (O-H), 2852–2923 cm^−1^(C-H), 1636.1cm^−1^ (C=C) and 1097.6 cm^−1^ (C-O), as depicted in Figure S1. These functional groups, including other functional groups, have been introduced during the synthesis process [19]. Oxidized MWCNTs have absorption peaks at 1741.1 cm^−1^ (Figure S1). This is due to the C=O stretching vibration from the introduction of carboxylic groups from the acids (HCl, H_2_SO_4_ and HNO_3_). A broader peak at 3441.2–3451.4 cm^−1^ in all the MWCNTs corresponds to the O-H stretch as a result of moisture in the MWCNTs (Figure S1). 

Only two peaks (3400 cm^−1^ and 1600 cm^−1^) were observed in all the MWCNTs under different treatments (including PEGylation) dispersed in water when scanned with UATR FTIR (Figure 4b). The universal attenuated total reflectance (UATR) accessory produces reproducible quality IR spectra of materials that polarize light using a variable-angle polarizer mount. Attenuated total reflection (ATR) enables materials to be scanned directly in the solid or liquid state without sample preparation. The peaks at 3400 cm^−1^ and 1600 cm^−1^ were due to the O-H (from water) and C=O functional groups in the CNTs, respectively. The disappearance of other peaks could be due to the low concentration of the treated MWCNTs in water [20]. 

MWCNTs in dichloromethane and the KBr power samples did not yield satisfactory results. These results confirm that the MWCNTs can be oxidized using acids and acylated using SOCl_2_. They also confirm that FTIR analysis of the MWCNTs using KBr pellets is the most appropriate technique, as opposed to KBr powders and an aqueous CNTs. 

UV-Vis spectroscopy measures the absorption of a beam of light after it passes through an aqueous sample or after reflection from a sample surface at a single wavelength or over the spectral range. UV-Vis spectroscopy was performed to confirm the functionalization of MWCNTs and removal of succinic anhydride following dialysis of DSPE-PEG5000-amine carboxylated with succinic anhydride. It was also performed to confirm the presence of the synthesized DSPE-PEG5000-4-arm (PEG-amine) in the preparation solution. DSPE PEG5000-4-arm (PEG-amine) was synthesized by reacting one equivalent (eq.) DSPE-PEG5000-amine (Nanocs Inc., New York, NY, USA) with five eq. succinic anhydride in dichloromethane (Sigma Aldrich Corporation, St. Louis, MO, USA). The reaction was stirred at an ambient temperature for 24 h and evaporated to dryness. The product was reconstituted in deionized water and dialysed against water in a SnakeSkin dialysis tubing (Pierce-MWCO 3.5 kDa, Thermo Scientific, Waltham, MA, USA) for two days at a neutral pH. The dialysis tubing was performed to remove the unreacted succinic anhydride (MW = 100.07 g·mol^−1^) from the product. Dialysis tubing content was lyophilized in a Virtis Freeze Dryer (Gardiner, NY, USA) for 24 h. Then, 1.5 eq. dicyclohexylcarbodiimide (DCC) was mixed with two eq. hydroxybenzotriazole (HOBt) in dichloromethane (DCM) purchased from the Sigma-Aldrich Corporation (St. Louis, MO, USA). The mixture was added to the lyophilized DSPE PEG5000-COOH and reacted at room temperature for an hour. The four eq. of 4-arm PEG-amine (Nanocs Inc., New York, NY, USA) was reacted with DSPE PEG5000-COOH in DCM for two days. The product was evaporated to dryness and reconstituted in deionized water and stirred for an hour at ambient temperature and filtered through a 0.22-m filter [21]. 

The completely dispersed CNTs are highly active in the 200 to 1200-nm wavelength region, and the UV-Vis technique is used to detect individual CNTs by relating the intensity of absorption at a specific wavelength to the concentration of CNTs suspended in the solution through the Beer-Lambert law. The highest optical wavelength (262.5 nm) was observed in SOCl_2_-functionalized CNTs and the least (198 nm) in HCl-functionalized CNTs (Figure 4c). These data are consistent with the findings of Tomova and colleagues [22]. Succinic anhydride is utilized in the synthesis of DSPE-PEG5000-PEG-4-arm amine. The unreacted succinic anhydride is separated from the product of interest via dialysis against neutral de-ionized water. It was detected in the dialysis buffer, as depicted by an optimal absorption at 200 nm (Figure 4c). 

The optimal wavelength at which pristine-aligned MWCNTs absorbed light was found to be 194 nm. Functionalization changed the optical behavior of the CNTs. In the HCl, H_2_SO_4_ and SOCl_2_-functionalized CNTs, optical wavelengths were 198 nm, 256 nm and 263 nm, respectively. The optical wavelength was shifted to the right of the pristine CNTs but within the UV range when CNTs were functionalized with HCl, H_2_SO_4_ and SOCl_2_, as depicted in Figure 4c. 

Optimal wavelength of pristine CNTs increased from 194 nm to 220.5 nm and that of HCl-functionalized increased from 198 nm to 220 nm, respectively, when they were dispersed in DCM. PEGylated CNTs’ optimal wavelength increased slightly from 194 nm to 196 nm (Figure 4d). The increase in absorbance could be due to the functional groups (carboxylic and acyl groups) that are added to MWCNTs and the size of the particles. These findings have demonstrated the effect of functionalization of the CNTs on their optical properties. The optimal wavelength increased with functionalization. Functionalization led to hyperchromic and bathochromic effects on the VA-MWCNTs, which could improve the sensitivity of the nanosensors for the detection of the biomarkers in several diseases. 

### 2.8. Thermal Properties of VA-MWCNTs

**Figure 5:**
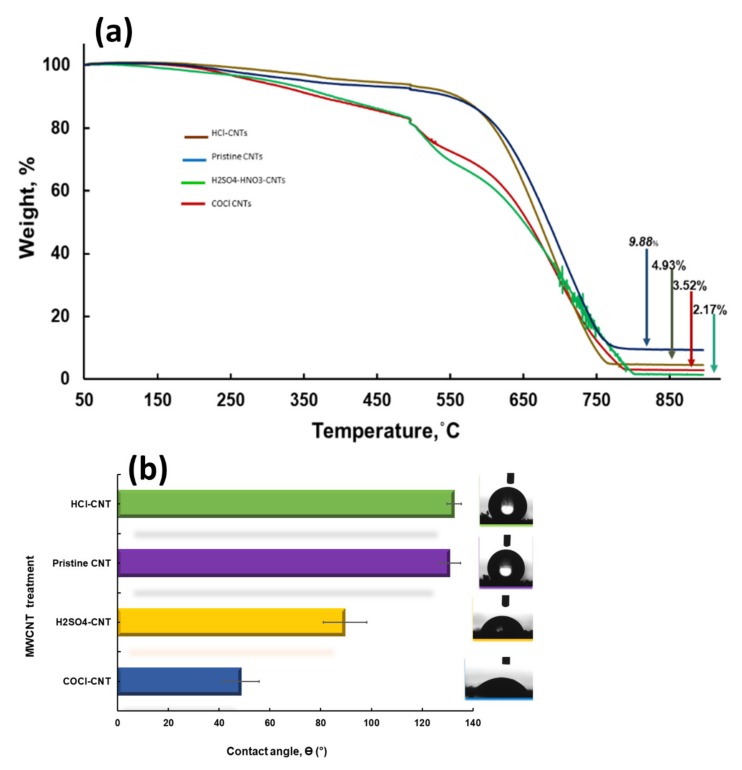
VA-MWCNTs properties. Thermogravimetric properties (**a**) and wetting properties (**b**).

TGA was performed in order to determine the thermal stability, degree of functionalization and purity of MWCNTs. The analysis of MWCNT powder was carried out under synthetic air flow, which implies that a thermal oxidation was investigated, and the residual at the end of thermal degradation was iron oxide. A minor weight loss observed from 50 °C to 150 °C corresponds to evaporation of the physi-adsorbed water from MWCNTs (Figure 5a). A gradual drop in weight from 150 °C to 500 °C is attributed to decomposition of MWCNTs as the temperature increases (Figure 5a). 

A drastic weight loss was observed around 550 °C in both pristine and HCl-MWCNTs (Figure 5a). In COOH-MWCNTs and COCl-MWCNTs, a drastic weight loss occurred around 500 °C (Figure 5a). A complete decomposition occurred around 830 °C in the pristine and functionalized MWCNTs, as confirmed by a change in color of MWCNTs from black to orange. Functionalization of MWCNTs with acids and SOCl_2_ purified the MWCNTs, as confirmed by less residual content in functionalized MWCNTs (2.17%, 3.52% and 4.93%), as opposed to pristine MWCNTs with a residual content of 9.88%, as depicted in Figure 5a. High percentage of the residue in pristine was due to the impurities, Fe nanoparticles and amorphous carbon. These findings have demonstrated the reduction in thermostability of the CNTs following the carboxylation and acylation of the CNTs. 

### 2.9. Wettability of VA-MWCNTs

Contact angle denoted θ is a quantitative measure of the wetting properties of a solid by a liquid. Contact angle is an angle which is formed when a liquid interacts with the solid at the three-phase boundary (liquid, vapor and solid) [23]. The contact angle below 90° is indicative of a hydrophilic material, whereas the contact angle above 90° is indicative of a hydrophobic material. The liquid wets the surface when the contact angle is less than 90°. The complete wetting occurs when the contact angle is zero. The material is nonwetting or waterproof when the contact angle is greater than 90°. The material is said to be semi-wetting when the contact angle is 90°. 

Contact angle measurements can be applied in carbon nanotubes following their synthesis for specific applications. The wettability of the carbon nanotube surface is a very important property that is influenced by both chemical composition and geometrical microstructure of the contact surface. In our work, drops of water were applied on the various spots of MWCNTs to measure their contact angles using a sessile drop technique [24]. The average contact angle for the HCl, H_2_SO_4_ and SOCl_2_-treated CNTs and for the pristine CNTs was 132.72° ± 0.98°, 89.58° ± 1.27°, 48.74° ± 2.79° and 130.88° ± 1.37°, respectively (Figure 5b). 

These results imply that the pristine and HCl-treated MWCNTs are hydrophobic, as their contact angles were found to be greater than 90°. The H_2_SO_4_-treated MWCNTs were semi-hydrophilic, as their contact angle was below 90°. The SOCl_2_-treated CNTs were hydrophilic, as their contact angle was significantly reduced to 48.74° ± 2.79° (Figure 5b). These results confirm the improvement of hydrophilicity of CNTs following carboxylation and acylation. It is very important to ensure that the carbon nanotubes should be hydrophilic, as they will be used in our future work as nanocarriers in delivering drugs to the diseased site in the animals. 

Contact angles were measured in order to determine the wettability of the MWCNTs. There is a direct relationship between the contact angle and hydrophobicity. When the contact angle is higher, the material under test is hydrophobic, which implies that there are less OH groups to bond with water molecules. The lower the contact angle, the higher the number of OH groups on the material of interest to bind to water molecules (Figure 5b). Application of carbon nanotubes in the biomedical field is a major challenge as a result of their hydrophobic nature creating low dispersity [25]. Drops of water were applied on various spots of the MWCNTs surfaces to measure their contact angles. 

Hoa characterized CNTs following treatment with different concentrations of H_2_SO_4_-HNO_3_ at different times. Optimization studies were performed to get the optimal conditions of CNTs’ functionalization with minimal or no defects in CNTs’ structures [19]. In our study, MWCNTs were grown on Si wafers treated with thermal and acid oxidation. They were found to be highly hydrophilic, as demonstrated in Figure 5b. 

### 2.10. Thermal and Oxidation Treatment of MWCNTs for Nanoelectrode Development

**Figure 6:**
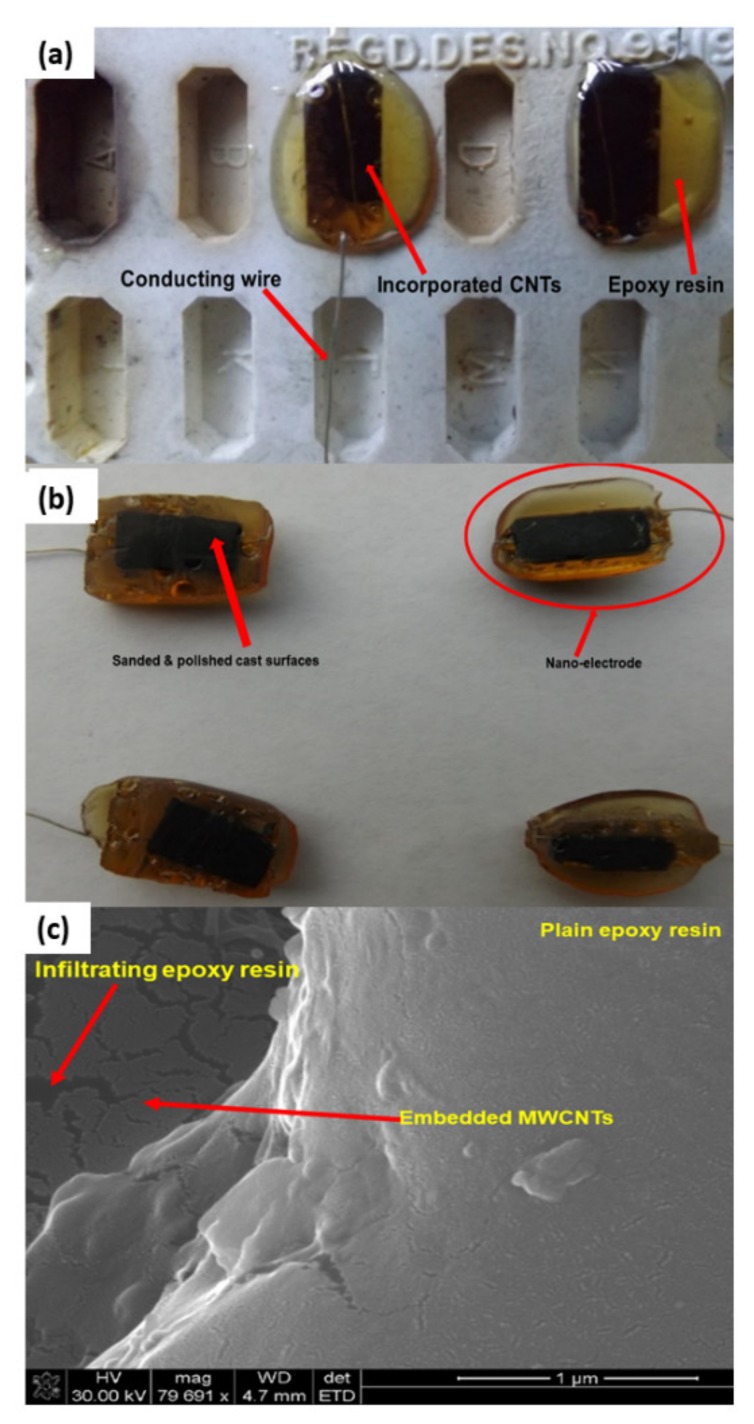
Incorporation of VA-MWCNTs in epoxy resin. Silicone rubber moulds for casting epoxy resin (**a**). Four sanded and polished nanoelectrodes (**b**). Epoxy resin with incorporated MWCNTs (**c**). CNTs are forming bundles in a vertical array (dark grey smooth surface), and the spaces between them are filled up with epoxy resin (dark cracks). The epoxy resin without CNTs is seen as a light grey solid lump without cracks (**c**).

Aligned CNTs were subjected to a temperature of 450 °C for 1 h at 200 mL·min^−1^ oxygen. They were then treated with HNO_3_:H2SO_4_ (1:3) and incubated for 2 h at 80 °C. MWCNTs were rinsed thrice with distilled water to remove the acid. MWCNTs were dried in a furnace at 450 °C for 30 min. These carboxylated silicon wafer MWCNTs were used to develop the nanoeletrode (Figure 6). 

### 2.11. Development and Morphological Assessment of the Nanoelectrode

Epoxy resin was used for the fabrication and manufacturing of the nanoelectrode in our study. Epikote 862 (diglycidyl ether of bisphenol F) is an epoxy resin produced from bisphenol F and epichlorohydrin. It contains no diluent nor modifiers. It possesses the following features: low viscosity; low color intensity; reacts with a full range of epoxy curatives; good balance of mechanical, adhesive and electrical properties; good chemical resistance and superior physical properties vs. diluted resins. 

Epoxy has been chosen as the polymer for our work due to its importance as the matrix of structural composites. The strong adhesiveness of epoxy originates from the polar bonds that the epoxy forms with the surface that it contacts. Epoxy is a thermosetting epoxide polymer that cures when it is mixed with a curing agent such as epicure 3402 (EC3402) (an amine acting as a hardener). An epoxy resin must be crosslinked in order to attain the desirable mechanical properties. 

A curing agent typically has active hydrogen atoms attached to nitrogen, oxygen or sulphur atoms. The reactive groups in the epoxy resin are mainly the terminal epoxide groups. Amine-curing agents are the most common for epoxy formulations. Amines are organic compounds that contain nitrogen as the key atom. Primary amines arise when one of the three hydrogen atoms in ammonia are replaced by an organic substituent, secondary amines have two organic substituents bound to nitrogen atoms, together with one hydrogen atom and tertiary amines have all three hydrogen atoms replaced by organic substituents. 

An organic compound with multiple amino groups is called a diamine, triamine, tetraamine and so forth. An amine-curing agent typically has more than three reactive sites per molecule. These sites facilitate the formation of a three-dimensional polymer network when the curing agent is mixed with the epoxy resin. The active hydrogen atom of the amine reacts with the epoxide group of the resin. The structure of the amine-containing organic compound and the number and type of amine groups in the compound govern the rate of crosslinking and the properties of the resulting polymer. 

In this study, Epikote 862 resin (bisphenol F-based epoxy) and Epikure 3402 (diethyltoluenediamine) were used to develop a carbon-based electrode. The resin and the hardener were chosen for their relatively low viscosity and long pot-life (several hours even at 80 °C), allowing sufficient time for processing and characterization of the samples. Carbon nanotubes need a mechanical support in order for them to be used as an electrode. CNTs were incorporated in the epoxy resin, and the surface of the CNTs covered by epoxy resin were exposed by sanding and polishing. The surfaces were washed with water to remove the loose epoxy resin from the surfaces of the CNTs (Figure 6b). The casts were further polished to expose the MWCNTs to improve the electrical contact. 

Furthermore, MWCNTs that were collected from quartz tube wall were placed near the magnetic stirring bar, and they were attracted to it. As-synthesized MWCNTs were attracted by a magnetic bar, indicating the presence of iron in the samples (Figure S2A–C). Magnetic properties are attained as a result of ferrocene used during CNT synthesis. The presence of iron in MWCNTs were confirmed by XRD analysis. The MWCNTs on Si wafer together with the electrical conducting wire were successfully incorporated in the epoxy resin, as shown in Figure 6a,b. 

Epoxy resin is one of the thermosetting polymers used in various industries, including aerospace, electronics and adhesive industries. Their application is based on their mechanical, chemical and thermal properties [26]. In this study, as-synthesized MWCNTs were incorporated into the epoxy resin to give them mechanical support. Epoxy resin-incorporated CNTs were exposed by sanding and polishing. They were then coated with Pd/Au and analyzed by SEM. The results obtained confirmed successful incorporation of the CNTs into the epoxy resin, as demonstrated in Figure 6c. Epoxy resin can be seen infiltrating between the CNTs. 

### 2.12. Electrical Conductivity of Silicon Wafer CNTs and Nanoelectrode

In order to understand electrical conductivity, it must be born in mind that CNTs can organize themselves into aligned bundles during their synthesis as a result of the presence of van der Waals forces. Purification, carboxylation, amination, PEGylation and any other form of functionalization have a greater impact on the electrical resistivity and conductivity of carbon nanotubes. The functionalization changes the physico-chemical architecture of the carbon nanotubes, hence, their electrical properties. 

**Figure 7:**
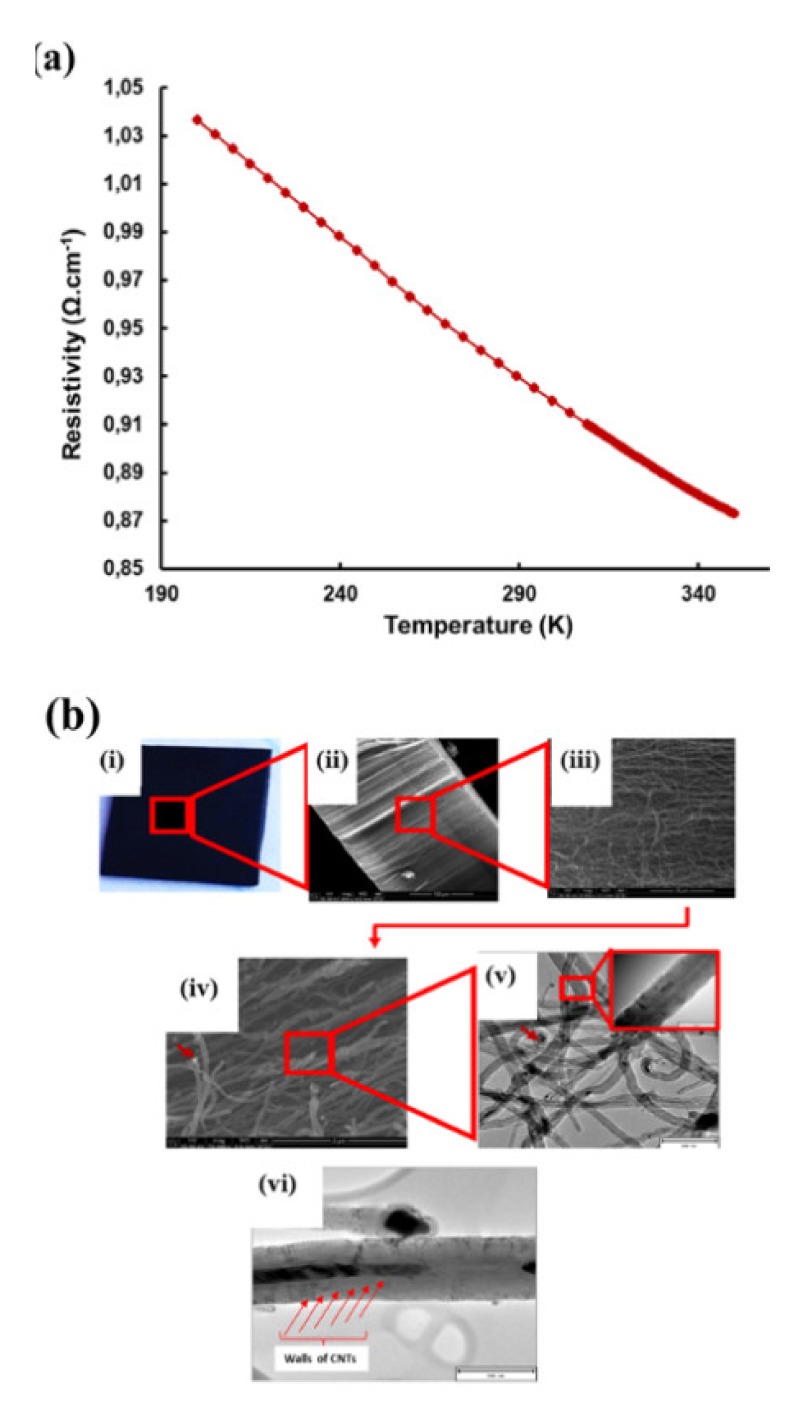
CNT electrical properties and MWCNTs from silicon wafer. Electrical resistivity of VA-MWNTs from 200 to 350 K (**a**). MWCNTS deposited on Si wafer (**b**(i)), SEM micrograph of CNTs at 844× magnification (**b**(ii)), SEM micrograph of CNTs at 8430× magnification (**b**(iii)), SEM micrograph of CNTs at 70,399× magnification (**b**)(iv) and TEM micrograph of CNTs with insert at very high magnification (**b**(v)). TEM micrograph of the MWCNT with red arrows indicating the location of the walls (**b**(vi)).

Qualitative and quantitative electrical conductivity assessments were performed prior to incorporation of the VA-MWCNTs into the epoxy resin for the development of a nanoelectrode. Further electrical conductivity measurements were done on the complete and fully developed electrode. The electrical resistivity (quantitative analysis) of the pristine VA-MWCNTs measured at various temperatures is depicted in Figure 7a. Resistivity of VA-MWCNTs was measured by PPMS from 200–350 K. As the temperature increased from 200 to 350 K, resistivity decreased, which implies that CNTS were electrically conductive. The electrical conductivity of the CNTs was improved at the temperatures from 310 K to 340 K, as depicted by the formation of the solid line in Figure 7a. 

Carbon nanotubes were grown on silicon wafer using CVD. A solution of 2.5% ferrocene in toluene was used as a catalyst. Vertically aligned multiwalled carbon nanotubes were successfully produced at 775 °C with a gas flow rate of 400 mL·min^−1^ for 45 min on both the silicon wafer and reactor wall. The pyrolysis conditions for the CNT growth was highly suitable for the growth of super-aligned MWCNTs on Si wafer in Figure 7b(i,ii). 

Super alignment was further confirmed at higher magnification, as depicted in Figure 7b(iii,iv). Fewer tips were still having Fe nanoparticles, as seen in Figure 7b(iv) (see red arrow). TEM scan further revealed the Fe nanoparticles embedded in the central cavity of the CNT in Figure 7b(v) (see red arrow). CNTs had more than one wall, hence, multiwalled CNTs, as depicted in Figure 7b(vi) (see red arrows). From these data, it can be concluded that the pyrolysis conditions were highly suitable for the growth of super-aligned MWCNTs with very few nanoparticles embedded in the central cavity of the CNTs. Our findings have demonstrated the high electrical conductivity of VA-MWCNTs. This property of MWCNTs enables them to be utilized in the development of a nanosensor for future diagnoses of a variety of diseases. 

### 2.13. Cyclic Voltammetry and Electrochemical Impedance Spectroscopy of a Nanoelectrode

Qualitative analysis of the nanoelectrode was performed by using electroanalytical techniques, namely cyclic voltammetry (CV) and electrochemical impedance spectroscopy (EIS), as described below. Electroanalytical chemical techniques help to analyze electrode-solution interfacial phenomena such as chemisorption, physisorption, charge transfer, chemical reaction and diffusion mass transport. An interfacial layer called a double layer is formed when an electrode is immersed in the electrolyte. This layer is formed between the electrode and the surrounding electrolyte as ions from the electrolyte are attracted to the surface of the electrode but leaving a very small gap between the electrolyte and electrode charges [26]. In a cyclic voltammetry (CV) setup, the working electrode potential is ramped linearly vs. time. Voltammetry applies a constant and/or varying potential at an electrode’s surface and measures the resulting current with a three-electrode system. This method can reveal the reduction potential of an analyte and its electrochemical reactivity. CV is most-commonly used, as it provides an overwhelming information and in-depth knowledge of both the kinetic and thermodynamic details of various chemical reactions [27]. 

Basically, CV is a linear potential waveform; the potential is changed as a linear function of time. In CV, the potential is measured over time, and the rate at which the potential changes with time is called the scan rate. The interface dynamics in an electrochemical system is measured by electrochemical impedance spectroscopy (EIS), which is phase-sensitive. A plethora of processes such as chemical reaction, mass transport and electron transfer have a greater impact on the output from an electrochemical impedance spectrometer. The EIS data is represented as Nyquist plots, where imaginary resistance (Z_im_) is plotted against real resistance (Z_re_). The size of the semi-circle is dependent of the resistance of the electrode. The bigger the diameter of the semi-circle, the higher the resistance, hence, the less the current passing through the electrode [28]. 

In our study, CV and EIS were used to analyze electrochemical properties of the developed nanoelectrodes using an Ivium Compactstat Electrochemical workstation. The electrodes were developed to be used in the preparation of nanosensors for the detection of biomarkers of a variety of diseases. 

**Figure 8:**
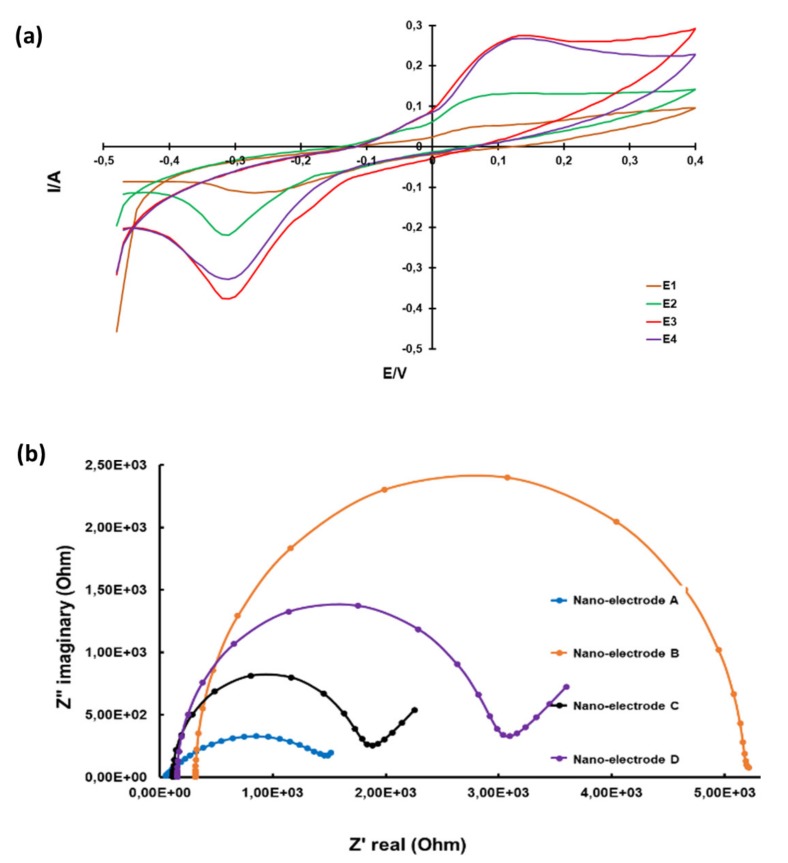
Electrochemical properties of MWCNTs. Cyclic voltammographs of nanoelectrodes (**a**). Nyquist impedance plots of nanoelectrodes (**b**).

CV data were recorded between −0.4 and 0.4 V with a scan rate of 50 mV·S^−1^ in a solution of 1-mM K_3_Fe(CN)_6_ and 1-mM K_4_Fe(CN)_6_ in 0.1-M KCl. Spectra were taken at a frequency of 100 kHz to 100 mHz with an amplitude of 0.01 V and potential of 0.220 V. All nanoelectrodes analyzed were found to be electrically conductive, with E1 being the least conductive and E3 the most conductive, as depicted in Figure 8a. 

EIS data confirmed the electrical resistivity of the nanoelectrodes. Nanoelectrode A was found to have the least electrical resistivity, hence, most electrically conductive. Nanoelectrode B resistivity was the highest, hence, least conductive, as demonstrated in Figure 8b. In this study, we successfully developed carbon-based electrodes which could be further improved for application in the detection of biomarkers of several diseases, including neurological disorders. 

Pristine carbon nanotubes are inert and hydrophobic. These properties hinder their application in biomedical fields. To employ them in a biomedical environment, they need to be functionalized. In this study, preformulations were performed on MWCNTs as a springboard for the development of a nanoelectrode and functionalization of MWCNTs for possible applications in the detection and treatment of a variety of diseases. 

### 2.14. Neurocompatibility of the MWCNTs

**Figure 9:**
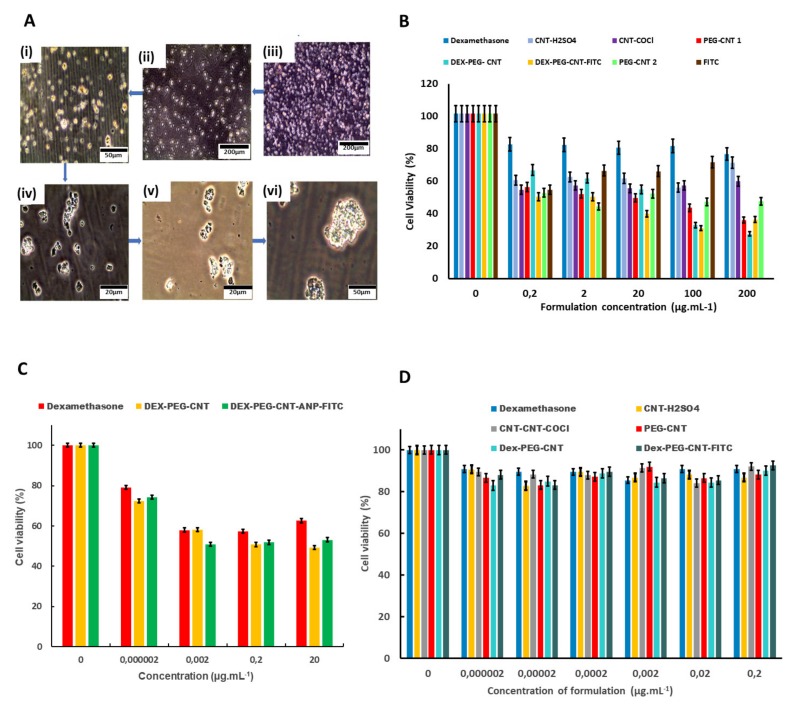
Cytotoxicity of the functionalized CNTs in PC-12 cells. PC-12 cells under inverted light microscope at different magnifications (**A**(i–vi)). Cell viability of PC-12 incubated with functionalized CNTs at different concentrations to 200 µg·mL^−1^ (**B**). Cell viability of PC-12 incubated with functionalized CNTs at different concentrations to 20 µg·mL^−1^ (**C**). Cell viability of PC-12 incubated with functionalized CNTs at different concentrations to 0.2 µg·mL^−1^ (**D**).

The PC-12 cells grew successfully with and without treatment with the formulations at various concentrations, as demonstrated in Figure 9A(i–vi). Only very few cells absorbed Trypan blue dye as a result of death, and this could be due to the formulations or other impinging factors, as the cells were not in their natural environment. The structure of the PC-12 cells could also be seen clearly under higher magnification (Figure 9A(iv–vi)). Cytotoxicity was determined by MTT assay (Figure S3A–D). The yellow MTT solutions formed blue formazan crystals in the live cells as the mitochondrial dehydrogenases cleaved the tetrazolium to insoluble formazan. The crystals were dissolved in acidified isopropanol (10% Triton X-100 plus 0.1-N HCl in anhydrous isopropanol). 

Different concentrations of the formulations were used as follows: high (0–200 µg·mL^−1^), medium (0–20 µg·mL^−1^) and low (0–0.2 µg·mL^−1^). The viability was 80–100% in the cells that were not treated with the formulations in all the concentration categories. The cell viability in the high concentration range was approximately 68%, 40%, 38%, 38%, 50%, 60% and 61% in dexamethasone, H_2_SO_4_, COCl, Dex-PEG, Dex-PEG-FITC, PEG and FITC-treated CNTs, respectively (Figure 9B). 

The cell viability in the medium concentration range was approximately 65%, 60% and 59% in dexamethasone, dex-PEG and dex-PEG-ANP-FITC-treated CNTs, respectively (Figure 9C). The cell viability was approximately 95%, 86%, 87%, 86%, 86% and 89% in Dex, H_2_SO_4_, COCl, PEG, Dex-PEG and Dex-PEG-FITC-treated CNTs in the low concentration range (Figure 9D). The cytotoxicity differed from formulation to formulation and from concentration to concentration. Our findings have demonstrated the reduction of cell viability with an increase in the concentration of the formulations. 

The reduced viability also depended on the type of the formulation. Cell viability was much better in the medium and low concentration range, as opposed to the high concentration range. Yuan and colleagues evaluated the effects of MWCNTs on macrophages and found that long MWCNTs were more toxic than short MWCNTs [29]. As these carbon nanotubes were suspended in culture medium and ultrasonicated prior to use, the toxicity could be due to the fact that CNTs were not functionalized. In our study, the CNTs were functionalized prior to use, and PC-12 cells were viable at the stipulated concentration range (more than 80%). 

## 3. Materials and Methods

### 3.1. Materials

The following chemicals were purchased from Sigma-Aldrich (Sigma-Aldrich Corporation, St. Louis, MO, USA): Ferrocene, 98%, toluene ChromaSolv for HPLC ≥ 99.9%, hydrochloric acid ≥ 32%, acetone ChromaSolv for HPLC ≥ 99.8%, ethanol 99.8%, bis-(cyclopentadienyl) cobalt(II) (colbaltocene) and bis-(cyclopentadienyl) nickel (II) (nickelocene). Circular silicon wafer with single-sided polish, N-type without dopant, diam. × thickness as 2 in. × 0.5 mm was also purchased from Sigma-Aldrich (Sigma-Aldrich, St Louis, Missouri, USA). Baseline argon (95%) balanced with 5% hydrogen was purchased from Afrox (African Oxygen Ltd., Johannesburg, Gauteng Province, South Africa). Copper grids with holey lacey carbon, Whatman filter paper and Pasteur pipettes were purchased from SPI supplies (SPI Supplies, West Chester, Pennsylvania, USA). Quartz reactor (1140 mm × 35 mm I.D.) was purchased from Wits (University of the Witwatersrand, Department of Chemistry, Johannesburg, Gauteng Province, South Africa). 

### 3.2. Instrumentation

A horizontal high temperature Elite TSH 12/38/500-2216E tube furnace (Elite Thermal Systems Ltd., Market Harborough, Leicestershire, UK) was used for the synthesis of carbon nanotubes. SEM analyses were carried out using FEI Nova NanoLab 600 FEG-SEM/FIB (FEI Company, Hillsboro, Oregon, USA). TEM analyses were carried out using FEI Tecnai T12 TEM. X-ray diffraction (XRD) studies were achieved using X-ray diffractometer (Philips, PANalytical X‘pert PRO, X-ray diffraction system) at 40 kV and 40 mA with Cu Kα radiation (1.54060) equipped with K-beta filter. Particle size distribution, polydispersive index and zeta potential measurements of the MWCNTs were carried out using a ZetaSizer NanoZS (Malvern Instruments, Malvern, Worcestershire, UK) instrument equipped with noninvasive backscatter technology set at a fixed angle of 173° and a light source with wavelength of 532 nm at ambient conditions. 

Thermogravimetric analysis of MWCNTs was performed by using a PerkinElmer TGA 4000, thermogravimetric analyzer (PerkinElmer Llantrisant, Wales, UK) with a temperature ranging from 30 °C to 950 °C under nitrogen gas. FTIR studies were done by Perkin Elmer Spectrum 2000 FTIR spectrometer (PerkinElmer Spectrum 100, Llantrisant, Wales, UK) at a resolution of 4 cm^−1^ with 10 accumulations with wavenumbers ranging from 4000–400 cm^−1^. Wettability of the CNTs was determined by the contact angle meter (Dataphysics, San Jose, California, USA). The UV-Vis spectra of the CNTs were measured by Shimadzu UV-2450 spectrophotometer (Shimadzu Corporation, Kyoto, Japan) at a wavelength range of 790 nm to 190 nm. Electrical resistivity of VA-MWCNTs was determined using a physical property measurement system (Quantum Design GmbH, Darmstadt, Germany). CV and EIS measurements were performed on the carbon-based electrodes using an Ivium potentiostat (Ivium Technologies, AR Eindhoven, The Netherlands). 

### 3.3. Preparation of Silicon Wafer and Catalyst

A circular silicon wafer (Sigma-Aldrich Corporation, St Louis, MO, USA) was cut into 1 cm × 1 cm pieces and sonicated in an ultrasonic bath (UMC-5, Integral Systems (Pty) Ltd., Johannesburg, Gauteng Province, South Africa) in acetone for 5 min followed by a second ultrasonication in water for another 5 min. The silicon wafer pieces were allowed to dry at room temperature. They were then stored at an ambient temperature in a dust-free environment for future use. 

For catalyst preparation, toluene was used as a carbon source and ferrocene as a source of iron nanoparticles for nucleation. Ferrocene catalyst was prepared by weighing ferrocene on ME 104 Mettler Toledo analytical balance (Merck (Pty) Ltd., Johannesburg, Gauteng Province, South Africa) and dissolved it in toluene. The solution was sonicated in a UMC- 5 ultrasonic bath for 15 min at room temperature to generate a homogenous mixture. 

### 3.4. Design and Optimization of Formulations using Box-Behnken Experimental Design (BBD)

Box-Behnken experimental design (BBD) was generated using Minitab^®^ V15 statistical software (Minitab^®^ Inc., State College, Pennsylvania, USA) to determine the optimal conditions for VA-MWCNT synthesis. The following variables were included in the experimental design: synthesis time, gas flow rate and synthesis temperature (Table 3). 

### 3.5. CVD Synthesis of VA-MWCNTs

The clean silicon wafer (1 cm × 1 cm) was weighed, placed in a quartz boat and loaded in a quartz reactor tube. The quartz tube was inserted horizontally in a horizontal high-temperature tube furnace. The ultrasonic nebulizer catalyst reservoir was filled up with 40-mL catalyst and attached to the gas line inlet, and the outlet was attached on the inlet of the quartz tube (Figure S3). 

A gas line with a mixture of 95% baseline argon balanced with 5% hydrogen was opened, and the mass flow controller (D08-4D/ZM, Beijing Sevenstar, Huachuang Electronic Co., Ltd., Dongguan City, Guangdong Province, China) was switched on, and flow rate was set to 50 mL·min^−1^. The Dr Hielscher UM20-1.6 MHz Sonic nebulizer (Hielscher Ultrasonics GmbH, Berlin, Teltow, Germany) was not switched on until the temperature reached the set point. Argon was used as a carrier gas to ferry argon into the furnace and hydrogen as a reducing gas to etch away amorphous carbon and impurities to produce clean CNTs. 

The outlet of the quartz tube was connected to the exhaust pipe. The furnace was switched on, and temperature was adjusted to 775 °C at a ramp of 10 °C·min^−1^. Flow rate was increased to 400 mL·min^−1^ once the temperature reached its set-point. The nebulizer was switched on for 45 min to generate an aerosol of catalyst. The aerosol that was generated was ferried into the high-temperature furnace by argon gas. All experiments were carried out at atmospheric pressure. The furnace was switched off, and flow rate reduced to 50 mL·min^−1^ following a 45 min synthesis to cool the furnace down to ambient temperature. Mass flow controller and gas line were switched off, and silicon wafer containing synthesized CNTs was collected from the furnace. CNTs were also collected from the quartz furnace tube by scrapping and weighed to determine the amount of CNTs deposited on the quartz tube wall. Precautionary measures were taken to collect only the CNTs’ halfway tube zone facing the nebulizer, since the centered samples were full of flakes and nanospheres. The collected samples were characterized as-synthesized using various techniques. 

### 3.6. Carboxylation, Acylation and PEGylation of MWCNTs

MWCNTs were purified by refluxing with HCl and oxidized by refluxing with H_2_SO_4_:HNO_3_ (3:1). An amount of 1.8 g of the HCl-purified MWCNTs was refluxed for 6 h at 70 °C in H_2_SO_4_:HNO_3_ (3:1). MWCNTs were then washed through centrifugation until pH was neutral. They were dried under vacuum at 70 °C for 24 h. Carboxylated MWCNTs were collected for characterization and further functionalization. Following carboxylation, MWCNTs were acylated with SOCl_2_. An amount of 1.0 g of MWCNT-COOH was added to 21.0 mL of SOCl_2_:DMF (20:1) mixture. The solution was refluxed for 48 h at 80 °C under nitrogen. The product was centrifuged at 5000 rpm for 15 min. Both the supernatant and precipitate were washed with THF using filtration via 0.22-µm filter and centrifugation respectively until the brown color due to SOCl_2_ vanished [21]. 

The COCl-MWCNTs were collected from the filters and centrifuge tubes and dried at 80 °C for 24 h using a dry vacuum oven (Trade Raypa, Terrassa, Spain). Acylated MWCNTs (0.2 mg·mL^−1^) were sonicated with 0.2-mM DSPE PEG5000-4-arm (PEG-amine) for 1 h. The product was centrifuged at 24,0000× *g* for 6 h using Centrofriger-BL11 centrifuge (JP-Selecta, Abrera, Barcelona, Spain), and supernatant was collected for characterization and further use. 

### 3.7. Dispersity, Particle Size and Zeta Potential of MWCNTs

The experiments were carried out by using functionalized MWCNTs mixed with different organic solvents in the concentration of 0.2 mg·mL^−1^. The different solvents used for dispersion were dichloromethane and deionized water. The pristine CNTs, H_2_SO_4_-CNTs, HCl-CNTs and COCl-CNTs were dispersed in deionized water and dichloromethane in 15-mL centrifuge tubes using an ultrasonic water bath and sonicated for 2 h. Dispersion of the CNTs in water and dichloromethane was observed visually and further confirmed using the dynamic light scattering technique. Measurements of the particle size distribution, polydispersive index and zeta potential of the dispersed functionalized MWCNTs were carried out using a ZetaSizer instrument at ambient conditions. 

### 3.8. MWCNT Morphological Evaluation

As-synthesized CNTs were peeled off from the substrate and prepared for SEM and TEM analyses in order to study their morphology and size. For SEM, CNTs were placed on a carbon tape or aluminum tape on the stub and coated with Pd/Au using Emitech K550X (Emitech Ltd., Canterbury, Kent, England) for 4 min at 25 mA and 2 × 10^−1^ mbar. For TEM, a small amount of CNTs was transferred into a 1.5-mL Eppendorf tube and dispersed in an absolute ethanol. The CNTs were sonicated in an ultrasonic water bath (Materials, Inc., Danbury, Connecticut, USA) for 10–15 min to disperse CNTs. 

Pasteur pipette was used to transfer dispersed CNTs onto a Cu grid that was placed on Whatman filter paper and was dried under light. The sample-loaded Cu grids were analyzed immediately or stored in vials for future analysis. Samples were loaded in SEM for the determination of morphology and length of the synthesized CNTs. SEM-EDS was used to determine the elemental composition of MWCNTs. Samples were also loaded in TEM for the determination of external and internal diameters of CNTs. ImageJ software was used to measure the length and diameters of the CNTs. 

### 3.9. Analysis of Crystalinity and Elemental Composition of MWCNTs

X-ray diffraction (XRD) patterns of the MWCNTs were examined on X-ray diffractometer, and each sample was scanned for 2 h because of high iron content in MWCNTs, and the raw data was processed with High Score Plus software. 

### 3.10. FTIR Spectra and UV-Vis of MWCNTs

KBr was dried and mixed with MWCNTs samples. The mixture was ground to fine particles and loaded on KBr die. The mixture was then pressed at 4-ton pressure under vacuum for 15 min with a KBr press (ICL, International Crystal Labs, Garfield, New Jersey, USA). The pellet was scanned with FTIR spectrometer. MWCNTs were also dispersed in water and loaded on universal attenuated total reflectance (UATR) to collect their spectra. Pristine and functionalized MWCNTs were dispersed in de-ionized water and DCM at a concentration of 0.2 mg·mL^−1^, and the spectra were measured by UV-Vis spectrophotometer. 

### 3.11. Measurement of Wettability of MWCNTs

CNTs were evenly spread on a thin double-sided tape on the sample platform of the contact angle meter. Drops of water were added at various spots on the pristine and 5-M HCl-treated CNT layers. Contact angles were measured using the sessile drop method and recorded. Snaps were taken for both the pristine and acid-treated CNTs. 

### 3.12. MWCNT Thermogravimetric Analysis

Thermogravimetric analysis of MWCNTs was employed to determine thermal stability of MWCNTs using a TGA4000 thermogravimetric analyzer (PerkinElmer Instruments, Llantrisant, Wales, UK). MWCNTs samples were subjected to a temperature range from 30 °C to 950 °C under a purging environment of nitrogen gas. The gas flow rate was set to 20 mL·min^−1^ and temperature ramp to 10 °C·min^−1^. Thermograms were generated as percentage weight loss vs. temperature. The raw data was processed using PyrisTM software (PerkinElmer, Llantrisant, Wales, UK). 

### 3.13. Carboxylation of Silicon Wafer MWCNTs for Nanoelectrode Development

Aligned CNTs were subjected to a temperature of 450 °C for 1 h at 200 mL·min^−1^ oxygen in a horizontal tube furnace. MWCNTs were transferred into a vial, and 3 mL of HNO_3_:H2SO_4_ (1:3) was added and incubated for 2 h at 80 °C in a Binder oven (Binder GmbH, Tuttlingen, Germany). MWCNTs were then rinsed thrice with distilled water to remove the acid. MWCNTs were dried in a furnace at 450 °C for 30 min. These carboxylated silicon wafer MWCNTs were used to develop the nanoeletrode. 

### 3.14. Development of Nanoelectrode

Epikote 862 (EK862) and epicure 3402 (EC3402) were dispensed into separate vials and incubated at 80 °C for one hour. An epoxy resin mixture was prepared by mixing EK862 and EC3402 in a ratio of 100:26.6, respectively. The mixture was further incubated at 80 °C for 30 min. The Si wafers with as-synthesized CNTs (purified and oxidized with acids) were placed in the wells of the silicone rubber moulds. Conducting wires were attached to the surface of the CNTs. 

Epoxy resin mixture was poured on top of the conducting wires, ensuring that air bubbles were not formed. The castings were incubated overnight at 80 °C to ensure that epoxy resin was cured properly. The casts were trimmed to remove excessive epoxy resin. 

### 3.15. Morphological Evaluation of Nanoelectrode

The embedded CNTs within the casts were exposed by fine sanding and polishing. The exposed CNTs were coated with Pd/Au using Emitech K550X (Emitech Ltd., Ashford, Kent, England) for 4 min at 25 mA and 2 × 10^−1^ mbar, and their morphology was studied by SEM. 

### 3.16. Electrical Conductivity of VA-MWCNTs and Nanoelectrode

Electrical resistivity of VA-MWCNTs was determined using the physical property measurement system (PPMS). The PPMS is an automated low-temperature and magnet system for the measurement of material properties like specific heat, magnetic AC and DC susceptibility and both electrical and thermal transport properties. In this study, VA-MWCNT with the dimension of 1 mm × 1 mm × 6 mm was loaded on the PPMS, and electrical resistivity was measured at temperatures ranging from 200 K–350 K. 

The surface of the CNTs covered by epoxy resin was exposed by sanding and polishing. The surface was washed with water and dried out. Magnetic bar was placed near the as-synthesized MWCNTs, and they got attracted to it. The casts were sanded to expose the MWCNTs to improve their electrical contact. 

### 3.17. Cyclic Voltammetry and Electrochemical Impedance Spectroscopy

Electrochemical measurements were conducted in a standard three-electrode cell, in which the MWCNT sites served as working electrode, platinum wire as the counter electrode and Ag/AgCl electrode served as the reference electrode. An electrolyte comprised of 1-mM K_3_Fe(CN)_6_ and 1-mM K_4_Fe(CN)_6_ in 0.1-M KCl. 

CV and EIS measurements were performed on the carbon-based electrodes using a potentiostat under ambient conditions. CV data were recorded between −0.4 and 0.4 V with a scan rate of 50-mV·S^−1^ in a solution of 1-mM K_3_Fe(CN)_6_ and 1-mM K_4_Fe(CN)_6_ in 0.1-M KCl. EIS was conducted under equilibrium conditions. Spectra were taken at frequency of 100 kHz to 100 mHz, with an amplitude of 0.01 V and potential of 0.220 V. 

### 3.18. Neurocompatibility Analysis of the Functionalised MWCNTs

PC-12 neuronal cells were used for evaluating the toxicity of the formulations prior to neuro-application. The formulations used in this study were as follows: dexamethasone (Dex), H_2_SO_4_-functionalized MWCNTs (H_2_SO_4_-CNTs), HCl-purified MWCNTs (HCl-CNTs), acylated CNTs (COCl_2_-CNTs), PEGylated CNTs (PEG-CNTs), dexamethasone-loaded PEG-CNTs (DEX-PEG-CNTs), FITC-labeled DEX-PEG-CNT (FITC-DEX-PEG-CNT) and ANP-coupled FITC-DEX-PEG-CNT (FITC-DEX-PEG-CNT-ANP). Cryopreserved PC-12 cells were grown in an incubator at 37 °C and 5% CO_2_ incubator in RPMI 1640 culture medium supplemented with glutamine, 5% fetal bovine serum (FBS) and 10% horse serum (HS). FBS and HS were inactivated by heating at 30 min at 56 °C in a water bath prior to introduction into the culture medium. A mixture of 1% penicillin and streptomycin (pen/strep) was added to the culture medium, and cells were checked on a daily basis for viability and microbial contamination by the Trypan blue exclusion technique. 

PC-12 cells were seeded in 96-well plates at a concentration of 6 × 10^4^ cells·mL^−1^ per well with the aforementioned formulations and incubated with various concentrations of dexamethasone and functionalized carbon nanotubes ranging from 0 to 0.2 µg·mL^−1^ in PBS (pH 7.4) for 24 h. Following incubation with various formulations, MTT assay was performed to evaluate the cytotoxic effect of the formulations at various doses. Samples were cultured in 96-well microplates and incubated with the MTT solution for approximately 4 h. Following incubation, a water-insoluble formazan dye was formed and solubilized by solubilization solution. The formazan dye was quantitated at 570 nm using Multilabel Reader (Victor X3, Perkin Elmer, Waltham, MA, USA). 

## 4. Conclusions

Vertically aligned MWCNTs were successfully synthesized using a chemical vapor deposition technique following optimization of the synthesis parameters, such as time, temperature, location of substrate in the reactor, catalyst and gas flow rates. Our novel findings include the successful development of a nanoelectrode which could be functionalized for future use in the detection of biomarkers in ischemic strokes and other neurological diseases. The hyperchromic and bathochromic shifts following the acylation and PEGylation of VA-MWCNTs could improve the sensitivity of the nanoelectrode for the biomarkers and reduction of opsonization of the CNT-based nanocarriers for targeted drug delivery. Furthermore, dispersibility, hydrophilicity and zeta potential of the VA-MWCNTs were significantly increased following carboxylation and acylation, and this could lead to improved stability and effective interactions of the CNTs with tissues in an aqueous biological milieu. The functionalized MWCNTs were nontoxic towards PC-12 neuronal cells. Moreover, the vertical super alignment of the MWCNTs could increase the sensitivity of the nanoelectrode for improved detection of the biomarkers. 

## Figures and Tables

**Table 1:** Thickness. Internal and external diameters of the vertically aligned-multiwalled carbon nanotubes (VA-MWCNTs).

ID	Rx	T (µm)	ID (nm)	OD (nm)	SEM	TEM
**30201406**	p CNTs	75	10	40	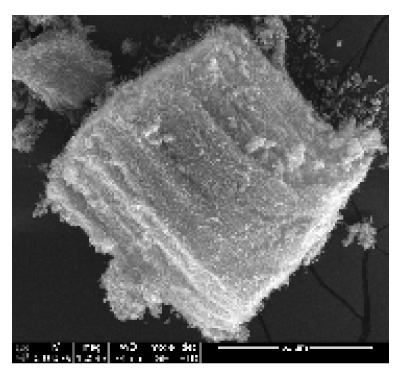	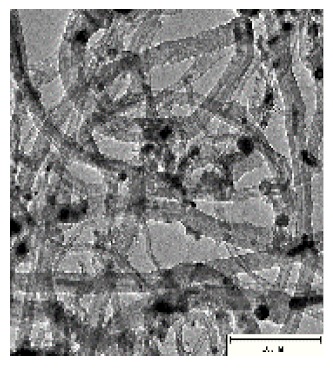
	HCl	75	8	34	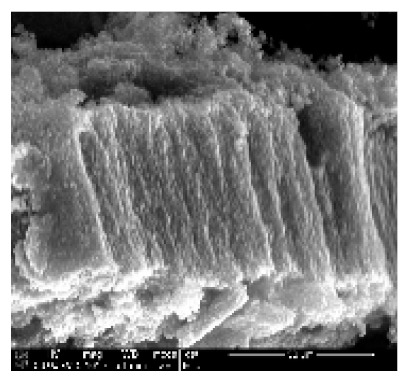	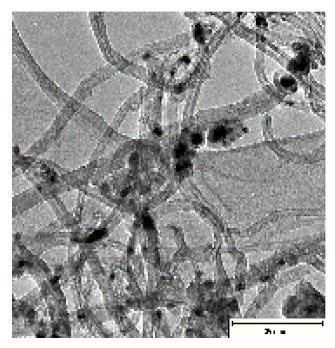
	H_2_SO_4_:HNO_3_	41	9	20	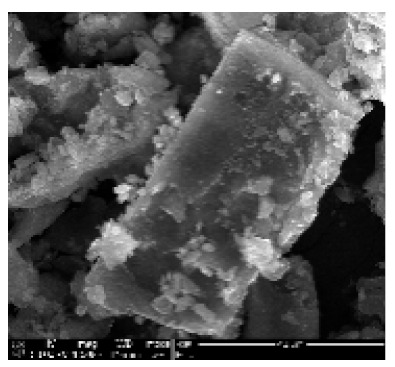	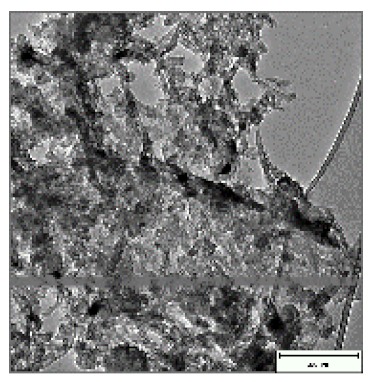
**04201406**	p CNTs	150	10	58	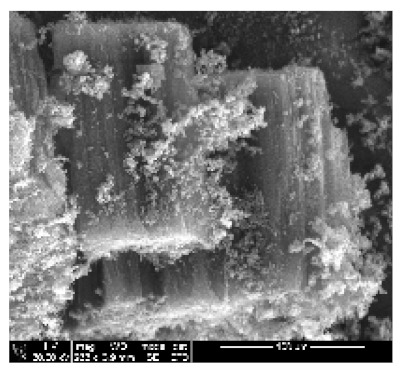	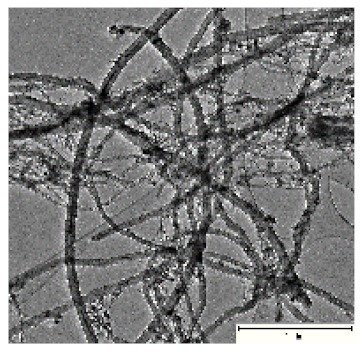
	HCl	140	11	46	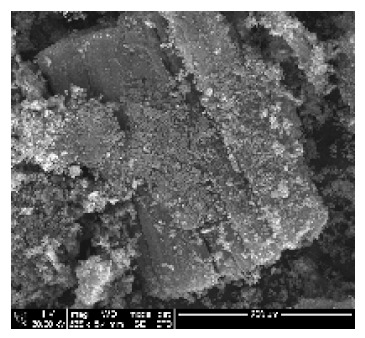	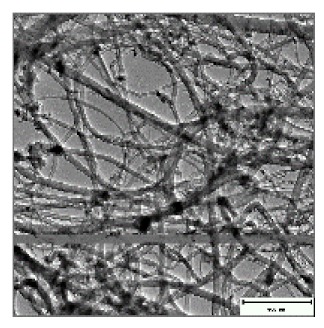
	H_2_SO_4_:HNO_3_	42	8	23	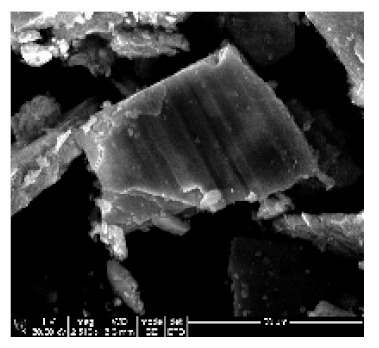	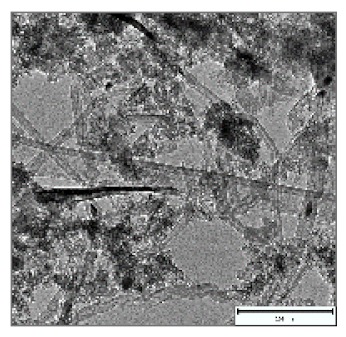

Legend: ID = sample ID, Rx = treatment, T = thickness, ID = internal diameter and OD = outer diameter.

**Table 2:** Particle size and polydispersive index of the functionalized MWCNTs in water and dichloromethane (DCM).

Functionalized MWCNTs	Particle Size (nm)	Polydispersive Index (PDI)
Pristine - water	320	0.39
Pristine - DCM	260	0.30
HCl -water	240	0.26
HCl -DCM	260	0.38
H_2_SO_4_ - water	250	0.34
H_2_SO_4_ - DCM	1200	0.40
SOCl_2_ - water	260	0.33
SOCl_2_ - DCM	6250	1.00
PEG - water	100	-

**Table 3:** Formulation variables as per the Box-Behnken design.

Formulation	Synthesis Time (min)	Synthesis Temperature (°C)	Gas Flow Rate (mL·min^−1^)
1	45	775	400
2	60	650	400
3	60	775	600
4	30	650	400
5	30	775	200
6	30	775	600
7	60	775	200
8	45	650	600
9	60	900	400
10	45	650	200
11	45	900	200
12	45	775	400
13	45	900	600
14	45	775	400
15	30	900	400

## Supplementary Materials

Supplementary materials can be found at https://www.mdpi.com/1422-0067/21/7/2276/s1.

## Abbreviations

VA-MWCNTs	Vertically aligned multiwalled carbon nanotubes
EIS	Electrical impedance spectroscopy
CV	Cyclic voltammetry
DCM	Dichloromethane
PDI	Polydispersive index
