# Supplementary material for: Functionalized, Vertically Super-Aligned Multiwalled Carbon Nanotubes for Potential Biomedical Applications
Source: Int J Mol Sci. 2020 Mar 25;21(7):2276. doi: 10.3390/ijms21072276 (PMC7178230; doi:10.3390/ijms21072276)

International Journal of
Molecular Sciences

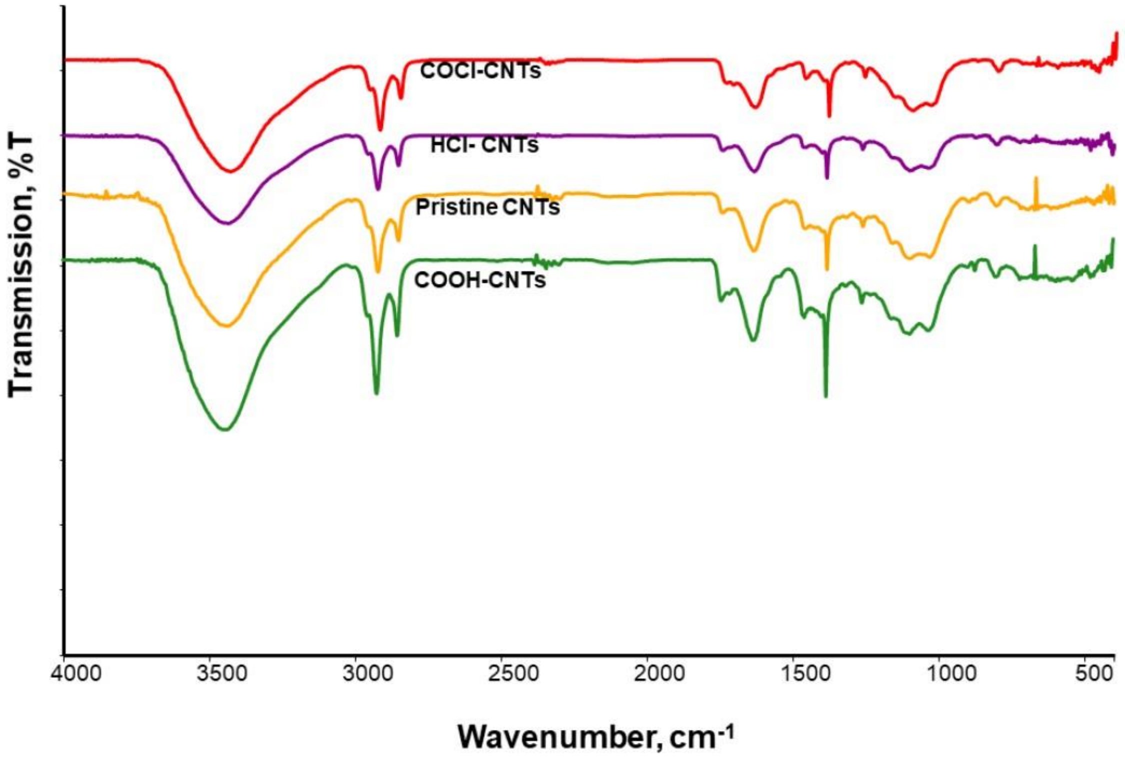

Figure S1. IR spectra of functionalised MWCNTs collected from KBr pellets.

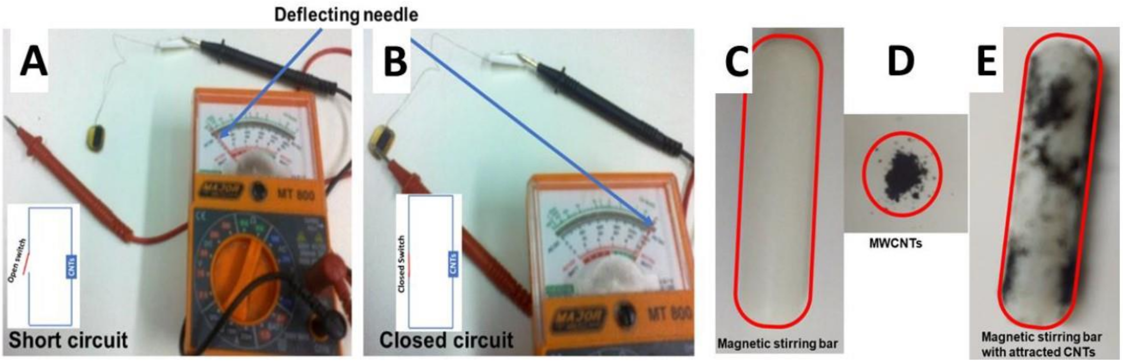

Figure S2. Magnetic properties of MWCNTs. Conducting meter with open (A) and closed circuit (B). Magnetic stirring bar (C). MWCNTs (D). Magnetic stirring bar attracting MWCNTs (E).

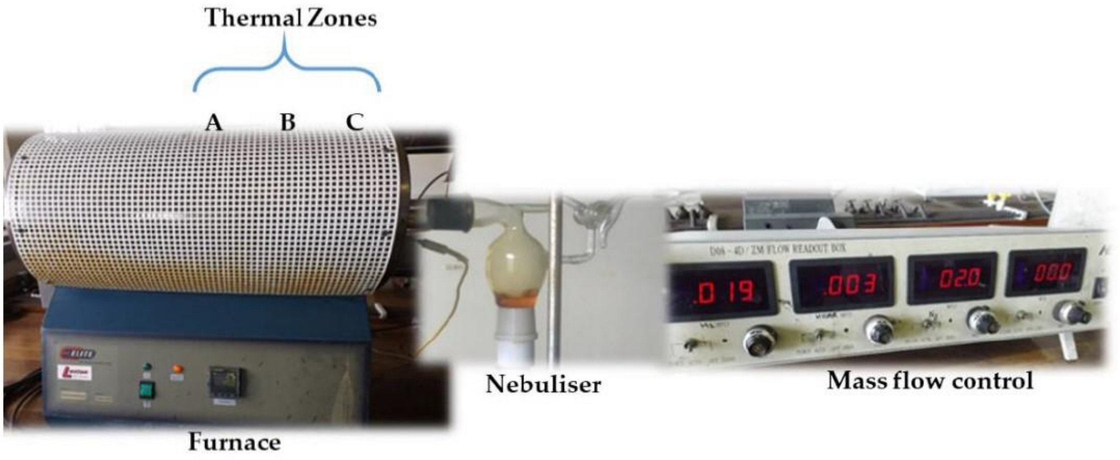

Figure S3. CVD set-up of the synthesis of VA-MWCNTs.

Table S1. BBDOE (A) Silicon Wafer and (B) Quartz Tube walls.

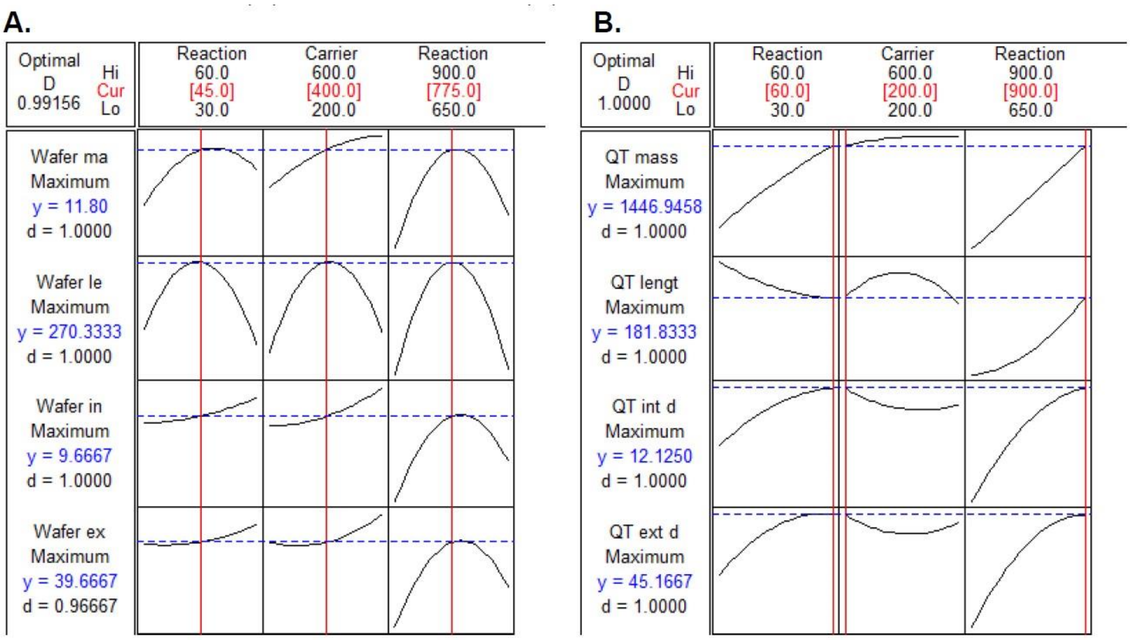

Supplement: Supplementary file 1 [file ijms-21-02276-s001.pdf]
